# Intense low-frequency sound transiently biases human sound lateralisation

**DOI:** 10.1371/journal.pone.0327525

**Published:** 2025-06-30

**Authors:** Carlos Jurado, Benedikt Grothe, Markus Drexl

**Affiliations:** 1 Audiology Group, Department of Neuromedicine and Movement Sciences, Norwegian University of Science and Technology, Trondheim, Norway; 2 Division of Neurobiology, Faculty of Biology, Ludwig-Maximilians-Universität in Munich, Martinsried, Germany; University of Central Florida, UNITED STATES OF AMERICA

## Abstract

Intense low-frequency (LF) sound exposure transiently alters hearing thresholds and other markers of cochlear sensitivity, and for these changes the term ‘Bounce phenomenon’ (BP) has been coined. Under the BP, hearing thresholds slowly oscillate for several minutes involving both stages of hyper- and hyposensitivity and it is reasonable to assume that the perception of sounds at levels well above threshold will also be affected. Here, we evaluated the effect of the BP on auditory lateralisation in healthy human subjects. Sound lateralisation crucially depends on the processing of either interaural level- or time differences (ILDs and ITDs, respectively), depending on the spectral content of the sound. The ILD needed to perceive a virtual sound source in the middle of the head was tracked across time. Measurements were carried out without and with a previous exposure to an intense LF-sound in the left ear, to elicit the BP. In 65% of the recordings, significant time-variant deviations from the perceived midline were observed after cessation of the LF-sound. In other words, a binaural stimulus perceived in the middle moved perceptually to the side and often back to the middle after presentation of the intense LF-sound. This means that intense LF-sound exposure can lead to a biasing of ILD-based sound localisation.

## I. Introduction

It has long been known that exposure to intense low-frequency (LF) sound (i.e., sound with spectral componentss < 200 Hz) can produce temporary changes in auditory sensitivity after cessation of the LF-sound, for a range of frequencies that extends well above the frequency of the LF sound [1–3]. This observation has been called the *Bounce phenomenon* (BP) and was first observed during the tracking of auditory threshold levels as a function of time, after an intense LF sound was presented. Soon after LF-sound exposure, hearing thresholds usually increase (desensitisation) and gradually return to pre-exposure levels within a couple of minutes, often after undershooting baseline threshold levels (sensitisation). Another sensation related to the BP is a usually roaring, tinnitus-like percept, also transient in nature and with a duration similar to other measures of the BP [4]. Objective biophysical measurements of cochlear properties, such as distortion-product otoacoustic emissions (DPOAEs), have also shown a BP in a manner consistent with hearing threshold shifts [5,6], even in the same subjects [4]. The combined evidence has led to the suggestion that the BP arises from temporary changes in the transduction of outer hair cells (OHCs), that provide active non-linear mechanical amplification and hence gain and compression [7–9]. To the best of our knowledge, no systematic study has been carried out exploring the effects of the BP on the perception of sound at suprathreshold levels. This is an important question, since it is unknown if the BP has the potential to impact everyday listening situations involving sounds which are typically well above threshold. Induced changes in OHC transduction, however, are expected to affect basic aspects of hearing, such as loudness perception. In turn, binaural sound processing depends on the activity level of inputs from the two ears. Changes in activity levels that may alter the perception of loudness are potentially also affecting binaural processing and therefore sound localisation or space-dependent sound source segregation.

Sound localisation at a fixed distance in the horizontal plane is dominated by interaural time differences (ITD) or interaural level differences (ILD), depending on the spectral content of the sound [10,11]. For pure tones in the sound field, humans use primarily ILD cues for frequencies above about 1.7 kHz, and ITD cues for frequencies below this [10,12]. Ambiguity in phase difference across ears limits the usability of ITD cues for pure tones of higher frequency, a range where corresponding physical ILDs become significant (due to multiple frequency-dependent refractive, reflective, and absorptive effects caused by pinna, head and torso; often referred to as head shadow [13]) and hence the dominant cue [10–12]. Even though pure-tone sound sources (at fixed distances) in the free-field produce increasingly smaller natural ILDs as frequency lowers, ILDs have been found to produce shifts in the perceived position of sound sources throughout the human hearing range [10] (with just-notable ILD differences being similar to those at higher frequencies, at least down to 200 Hz [14]). In addition to ITD and ILD cues, spectral cues are used to discriminate sounds with ambiguous ITDs and ILDs, such as sources in the median plane [10,15].

Effects of altered binaural cues on sound localisation have been mainly explored with plugging one ear in participants with normal hearing (thus simulating a conductive hearing loss [16,17]), and it has been shown that adaptation to these new binaural cues can restore horizontal sound localisation to some extent, probably by relying on monaural spectral cues [18]. Taken together, it seems plausible that the BP can cause a transient, dynamic change in the perception of ILDs, resulting in biased sound lateralisation and sound localisation.

Thus, the main aim of this work has been to evaluate whether the BP can significantly affect ILD perception, resulting in biased sound lateralisation.

## II. Methods

The experiment consisted of two parts: (1) tracking of ILDs required to perceptually centre a sound image over time and (2) tracking hearing thresholds over time. Part (1) was further divided into two sessions: a screening session, and a data collection session. Part (2) was done in a single session. Each session was performed on a different day, and sessions lasted between 1.5 and 2 hours.

### A. Sound lateralisation

#### 1. *Rationale.*

Human sound localisation in the horizontal plane depends mainly on ILDs (defined as the difference between the left- and right-ear sound pressure levels) and ITDs (difference between left- and right-ear sound arrival times). If a sound has a higher level at the left than at the right ear, the sound source will appear to be located towards the left side, and *vice versa* if the sound level is higher at the right ear. Similarly, if the sound arrives earlier at the left or right ear, the source will appear to be located towards the left or right side, respectively [10]. In this work, test signals were presented without delays between the ears, resulting in no ITDs, but the ILD of binaurally presented stimuli was varied to make their source image shift both towards the left (defined as increasing or positive changes in ILD) and towards the right (defined as decreasing or negative ILD changes) sides of the head. By monitoring the ILD that made sound probes laterally shift a fixed perceptual amount to each side of the head (see details below), the ILD corresponding to the ‘centre of the head’ position was tracked as a function of time. The latter will be referred to as centering ILD (cILD). It was assumed that if one of the ears presented temporal alterations in sound intensity encoding (i.e., was under the BP), the relation between source image and ILD would be different and vary across time, leading to a cILD deviating from control conditions. Therefore, a cILD tracking task was performed both without and with a previous exposure to an intense LF-sound.

#### 2. *Procedure.*

Clearly audible sinusoidal sound probes (40-Phons level according to ISO-226 [19]; as presentation was via insert earphones, a compensation for the free-field to eardrum pressure transfer function was further applied according to [20]) were presented binaurally via insert-earphones to the subjects and the ILD was varied. The procedure was analogous to a Békésy tracking [21] (also used here for hearing threshold measurements, see section II.B), where instead of the stimulus level as a parameter, the stimulus ILD was varied from 0 dB to negative and positive values, as a function of time. This caused the sound source image to perceptually move laterally, specifically from left-to-right or right-to-left when the ILD decreased or increased, respectively. The ILD was varied in 2-dB steps, by increasing/decreasing the sound level in one ear by 1-dB while decreasing/increasing it the same amount in the other ear. The chosen ILD step size was about 1 dB above the just-notable-difference for ILD changes reported in the literature (0.5–1 dB [11]). The probe tones had a 250-ms duration (with 100-ms ramps at start and end; “half-height” duration of 150 ms) and were presented in sequence, being separated from each other by a silent period of 150 ms. The ILD of subsequent tones in the sequence was continuously increased or decreased, with the direction of change (increase or decrease) being controlled by the participants. To make the sound image move to the left, participants pressed the left-side button of a gamepad (Xbox 360; Microsoft, USA) and to make it shift to the right, they pressed the right-side button. The initial ILD of a run was set to 0 dB and its initial direction of change (increase or decrease) was set at random.

**Fig 1:**
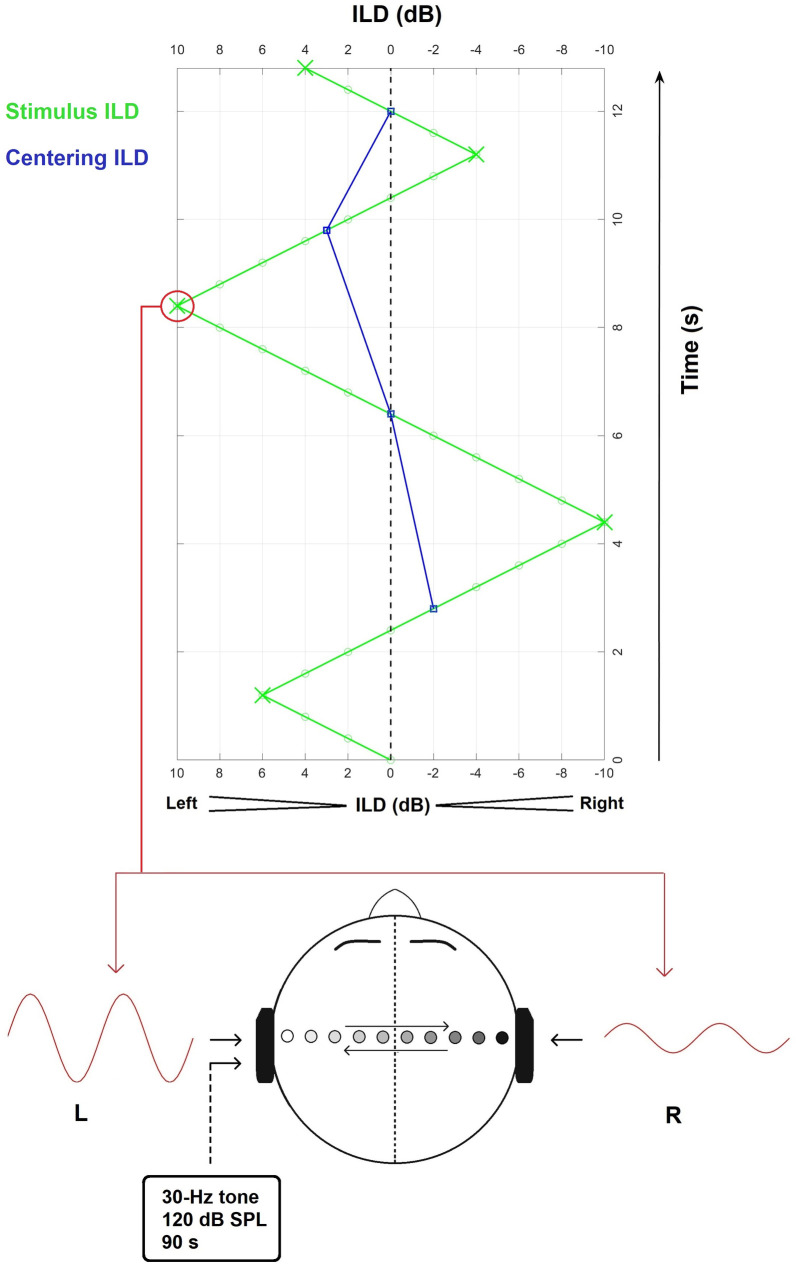
cILD tracking procedure. The stimulus ILD was adjusted by changing the relative levels of the left- and right-ear signals (L and R, respectively) in opposite directions, which made the sound source image perceptually shift from left-to-right or right-to-left (see filled circles and arrows inside the head). The displayed wave amplitudes are shown for an instance when the stimulus ILD (green squares) was 10 dB. The ILD of subsequent binaural probe tones was increased in a given direction (see x-axis) until the subject pressed a button, shifting the direction of change (green crosses denote turnpoints). The average of subsequent ILD turnpoints defined the “centering ILD” (blue). The tracking was carried out both without and with previous exposure to an intense low-frequency stimulus on the left ear.

Participants were instructed to let the perceived sound image shift towards one side of the head and then towards the other side, after passing through their perceived centre of the head. The amount of shift was a subjective point where the subjects clearly perceived the sound was at a given side of the head. For ease, the latter will be called point of subjective laterality (PSL) and corresponds to a turnpoint or excursion in the Békésy tracking. Participants were further instructed to aim for the same PSL cue, regardless of which side of the head the sound image was moving towards. The cILD was then obtained from averaging subsequent PSLs. Fig 1 illustrates the cILD tracking procedure.

To establish a baseline cILD pattern, the lateralisation task was first performed for 120 s without LF-sound exposure. Following this, the task was performed again for 240 s, immediately after exposure to an intense LF stimulus. The latter consisted of a 30-Hz tone at 120 dB SPL (80.5 dBA), presented at the subject’s left ear for 1.5 minutes. Lateralisation measurements (no stimulation followed by LF-stimulation conditions) were performed for probe-tone frequencies of 0.5, 1, 2 and 4 kHz. The order of probe-tone frequencies was randomised.

A preceding screening session dedicated to training (1.5 hours) was performed so that subjects would learn to recognize and repeatedly track their PSL for each probe-tone frequency. Subjects that showed evident difficulty in reaching a stable cILD baseline pattern (e.g., the baseline increasingly drifted to one side, easily recognized by the experimenter) were not invited to the subsequent two data collection sessions and were excluded from the experiment (i.e., no cILD nor threshold data was collected from these subjects).

### B. Sound detection

In another session, detection thresholds were tracked in time, to evaluate whether subjects presenting temporary changes in laterality perception would also experience well-known temporary threshold shifts after intense LF-sound exposure. Similarly as done for the cILDs, a Békésy tracking [21] procedure was employed, where subjects controlled the direction of probe-tone level change. By pressing one button of the gamepad, subsequent probe-tone levels continuously decreased until the probe tone was rendered inaudible. At this point, subjects pressed another button so that the following probe-tone levels would increase until the sound probe was audible again, and so on. A 20-min training block took place so that subjects familiarized themselves with the task.

Sound probes had the same characteristics as in the sound laterality tests (see section II.A.2), excepting that they were presented monaurally in the left ear (the LF-stimulated ear). The level of the subsequent tones in the sequence was changed in 2-dB steps.

Prior to the LF-sound exposure, a baseline threshold was measured for each subject with the Békésy tracking. Here, the starting level was set at 25 Phons and the initial direction of change was decreasing sound level. The measurement lasted 120 s and a representative threshold value (for later use) was obtained from averaging 95% of all the turnpoints, excluding the initial ones.

Following this, the LF-sound exposure took place and immediately after it stopped, the detection threshold was tracked again with the Békésy procedure for 240 s. The initial probe-tone level was set at the subject’s baseline threshold and the initial direction of level change was positive (i.e., increasing sound level). The sound detection task was performed for the same probe-tone frequencies as the lateralisation task, in randomized order.

### C. Apparatus and calibration

Experiments were controlled and monitored using custom-made programs in MATLAB (version R2023a, The MathWorks, Inc., Natick, Massachusetts, US). The binaural tones whose ILD was varied were generated using two ER-10C in-ear receivers (Etymotic Research Inc, Illinois, USA; one for each ear) and the low-frequency stimulus was delivered using an NSW1-205-8A 1-inch speaker (Aurasound Inc., USA), adapted for the playback of low-frequency sound. The ER-10C receivers were driven by a Fireface UC multichannel sound card (RME Audio, Haimhausen, Germany), run with a 48-kHz sampling rate and 24-bit resolution. The low-frequency speaker was driven by a NAD 312 power amplifier (NAD electronics, Toronto, Canada), itself connected to a channel of the soundcard. The acoustic output of the adapted speaker was delivered to the subject’s left ear via a long thin polythene tube, going through a piercing of the ER-10C earplug.

To ensure correct sound levels, the frequency responses of both ER-10C microphones and receivers, and the adapted low-frequency speaker, were measured. The reference microphone was a B&K ear simulator (type 4157; Brüel & Kjær Sound and Vibration Measurement A/S, Denmark), connected to a measuring amplifier (B&K type 2636). A 20-s long white noise signal was generated, and after time-domain averaging (50 buffers, 400-ms long each), responses were obtained via frequency domain deconvolution (2.5-Hz resolution). Absolute levels were determined from recording the acoustic signal (1 kHz at 94 dB SPL) from a sound calibrator (Nor1256; Norsonic AS, Tranby, Norway).

*In situ* calibrations were also performed to ensure correct sound levels were delivered at the individual ear canals. These were done with the ER-10C microphone (its frequency response was compensated using frequency domain deconvolution) fitted to the ear canal via the earplug, using the same sound signal as described above. Calibration was done after each fitting of the left and right ER-10C in-ear probes to the subject’s ears and after every other psychoacoustic measurement run. Besides the fix provided by the earplug fitting, the in-ear probes were fastened in place using surgical tape, minimising slippage. To minimize contamination of the *in-situ* calibrations with unwanted noise (e.g., due to the subject moving), an artifact rejection routine was implemented. Here, time-domain buffers with outlier powers (more than 3 scaled-median absolute deviations from the median [22]) were excluded from the time-domain averaging. The latter was a weighted average, where the sum of weighted buffers -with weights inversely proportional to their power- was divided by the sum of all weights [23].

The *in-situ* calibration routine was tested by providing different fittings of the ER-10C in-ear probes to the B&K 4157 ear simulator and playing back the probe tones at desired levels, which were confirmed with the B&K 2636 measuring amplifier.

### D. Data analysis

The average of successive turnpoint pairs was used to estimate the cILD, that corresponded to the centre-of-the head position at a given time. The corresponding time was set to be the average time between the two successive turnpoints, with the two first turnpoints being excluded. The same was done for the detection threshold data, only that turnpoint pairs corresponded to successive excursion levels in the Békésy tracking. The statistical analysis of significance described below was performed on the “raw” time-series data (cILDs and thresholds) defined in this manner. The complete dataset can be found in the supplementary material. Mean cILDs and thresholds for each probe-tone frequency were then obtained from interpolated versions of the individual curves (1-s steps), after restricting them into a common time duration range where all presented data. In the figures, individual and mean curves have been smoothed for visualization purpose (a five-turnpoint moving average was applied to the individual curves and a 25-s moving average to the mean curve; the latter corresponded to five turnpoints on average). A damped harmonic oscillator model was fitted to the unsmoothed average cILDs for each probe-tone frequency, where amplitude, damping, period, and phase were free parameters.

To evaluate whether changes in the cILD (and threshold) baseline and LF-stimulation conditions were occurring randomly or systematically, a change-point analysis algorithm was applied [24]. The approach utilizes cumulative sum analysis (first introduced by [25] to detect change points; for reviews see [26,27]) in combination with bootstrapping (initially proposed in [28]; for a review of bootstrap methods, see [29]). The excursion, defined as the difference between the maximum and minimum of the cumulative sum of the time series was obtained for the original time series, after subtracting it by its mean value. The original excursion was compared to that obtained with identical analysis performed on a large number of new time series (L = 10000), generated by moving-block bootstraps (MBB) of the original one [30–32]. Overlapping blocks of length *K* = 2 were used. This was done to account for expected nearby-dependency in turnpoints derived from the Békésy tracking, where the previous excursion level might influence the next. The proportion of excursions of the MBB cumulative sum distribution that fell above the observed difference defined the probability of occurrence of the latter. The significance level for both the baseline and LF-stimulation conditions was set at 1%. This means that the passing criterion for the BP required that both *p* > 1% for the baseline (i.e., non-significant excursion) and *p* < 1% for the LF-stimulation condition (significant excursion due to a change point).

To examine whether cILDs measured after the LF-stimulation condition fell markedly above or below the baseline cILDs, result figures include lower and upper outlier limits of the corresponding baseline cILD distribution. The latter were respectively defined as Q_1_ - 1.5 × IQR and Q_3_ + 1.5 × IQR (where Q_1_: 1^st^ percentile, Q_3_: 3^rd^ percentile and IQR: interquartile range [33]).

To evaluate the shape similarity across individual cILD curves and find the most representative individual data for each probe-tone frequency, the cross-correlation between time series (evaluated at zero time lag) was used as parameter. Prior to this analysis, individual cILDs were restricted into a common time duration range where all presented data points, a five-turnpoint moving average was applied and the curves were interpolated into 1-s steps. All comparisons provided a 17x17 matrix of cross-correlation coefficients that were arranged in descending order for each row and hence the 2^nd^ column contained the most similar curve relevant for each case (the 1^st^ column corresponds to trivial self-comparison). The most representative individual curve was defined as that most frequently presenting the highest similarity to all others, i.e., the mode of the 2^nd^ matrix column.

To account for the different BP patterns observed in cILDs and thresholds, a simple model described in [4] was used. This qualitative model evaluates the effects of time-varying operating point (OP) shifts on OHC mechano-electrical transduction (MET). The transfer function of the OHC MET (as described in [34]) was used to simulate BPs of both threshold and cILD. It was assumed that increased OHC efficiency leads to larger sound-evoked activity levels, and further that the cILD is reached when left and right input levels are adjusted so that equal internal activity levels occur in both ears [35,36]. Oscillatory OP changes were simulated with different damping and periods, to broadly reflect BP trends that were observed for the lower and higher probe-tone frequencies. The initial movement of the OP shift was assumed to be in the hyperpolarizing direction (as observed in [37]; however, analogous results can be obtained by inverting the OP shift direction and its initial position relative to the inflection point on the MET transfer function).

### E. Subjects

A total of 31 participants (18–50 yrs) were initially recruited for the experiment. Fourteen of these participants either did not present consistent responses in the baseline laterality task (n = 8), presented a hearing loss (n = 5; HL > 20 dB, determined via a pure-tone audiometry for the probe-tone frequencies and the 30-Hz tone), or decided not to attend the following sessions (n = 1). This left 17 subjects (18–45 yrs; mean: 31.6 yrs; 11 females and 6 males), of which 16 presented data across the 3 sessions (one participant did not attend the detection threshold session). Participants were compensated for taking part in the experiments. The experiments are in accordance with the declaration of Helsinki and were approved by the Norwegian Regional Committee for Medical and Healthcare Research Ethics (project ID: 704549). The recruitment period started on 14.03.2024 and ended on 31.05.2024. Written informed consent was obtained from all participants.

## III. Results

**Fig 2:**
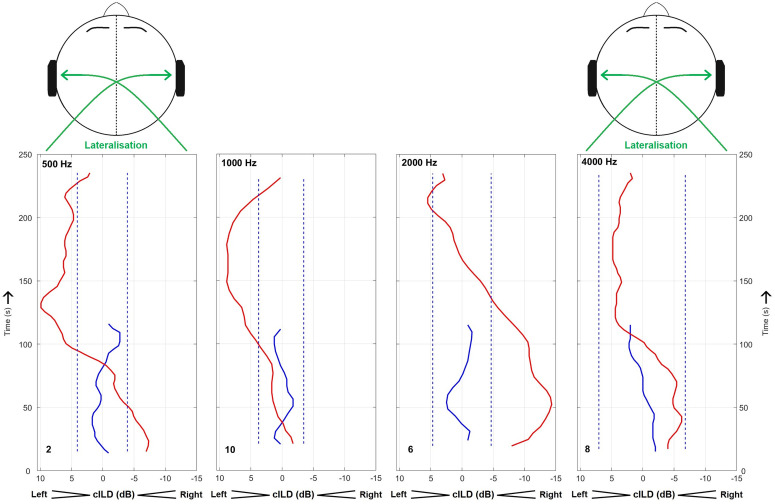
Representative examples of interaural level differences required to centre a sound source image (cILDs). In each case, the cILDs obtained before (blue) and right after exposure (red) to an intense low-frequency stimulus are shown. For each probe-tone frequency (upper left), the most typical pattern is shown (see subject ID in bottom left). As time progressed (vertical axis), systematic changes were observed in the cILDs after the low-frequency sound exposure. The left and right vertical dashed lines are respectively upper and lower outlier limits of the baseline cILD distribution (see data analysis for details). For ease in the comparison, the across-time average of the baseline cILD was subtracted from the baseline and LF-stimulation cILDs (individual and mean un-normalized cILDs are shown in Fig 4). Relative to baseline levels, positive cILDs correspond to higher level in the left-side probe tone and *vice versa* for negative cILDs (see labels on the horizontal axis). Under the BP, perceived lateralisation of the sound image for a stimulus presented with baseline ILD (zero normalized ILD) is in the opposite direction to the cILD, as shown in the above diagrams.

In the majority of cases, we observed a significant BP in the time series of the ILDs required to centre the sound source (65% across all conditions according to our passing criterion; 59, 59, 65, and 77% of cases for 0.5, 1, 2 and 4 kHz, respectively). In 61% of the hearing threshold measurements, significant transient changes in accordance with the BP were observed (75, 75, 50 and 44% of cases for 0.5, 1, 2 and 4 kHz, respectively). According to our passing criterion, most significant BPs were determined by whether or not the LF-stimulation condition induced a BP, as the majority of subjects presented stable baselines (cILD: 98.5%, threshold: 81.3%). Among the subset of subjects that presented a significant BP in their cILD, 54% of them also presented a significant BP in threshold (67, 67, 40, and 42% of cases for 0.5, 1, 2 and 4 kHz, respectively). The most typical cILD patterns, shown in Fig 2 for each probe-tone frequency, start with negative values (higher level required for the right-side tone), which then go back to baseline levels or go through a positive region (higher level required for the left-side tone) before returning to baseline levels. The occurrence of a positive cILD region beyond the baseline range was most often observed for the lower probe-tone frequencies than for the higher ones (observed in 76.5%, 64.7%, 41.2% and 41.2% of individual cases for 0.5, 1, 2 and 4 kHz, respectively). This contrast is most evident in Fig 2 comparing the leftmost with the rightmost panel. There was no systematic dependence on probe-tone frequency in the span (average difference between subsequent turnpoint levels for a run) of their cILD tracking (the across-subject average span was 24.1, 24.4, 24.2, and 24.9 dB for 0.5, 1, 2 and 4 kHz; ANOVA: F_3,64_ = 0.074; *p* = 0.97).

**Fig 3:**
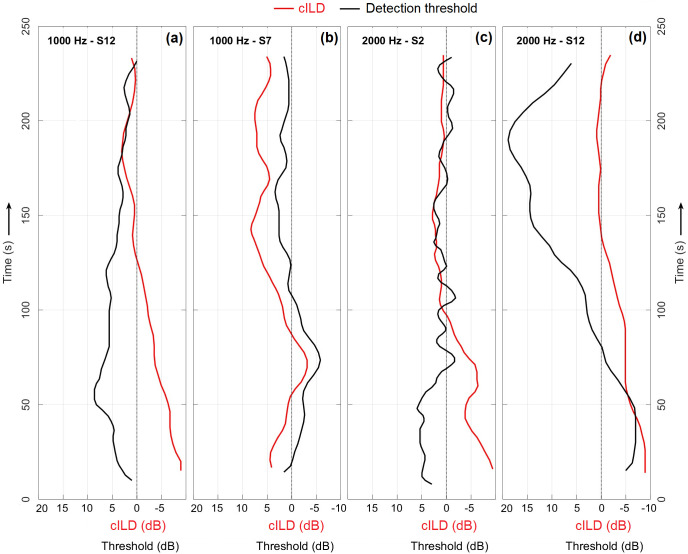
Example individual data (see details in upper left) that presented a significant BP in both cILD (red) and detection threshold (black). Both mirror-inverse (panels **(a)** and **(c)**) and symmetrical patterns (panels **(b)** and **(d)**) could be identified among the subset of cases that presented a valid BP in both measures. To facilitate the comparison, cILDs and thresholds were subtracted by the across-time average level of their corresponding baseline.

As has been observed previously, detection thresholds after LF-sound exposure usually presented a desensitisation that came soon after exposure (e.g., see black lines in Fig 3, panels (a) and (c)). More complex oscillatory patterns were also observed, such as initial sensitisation followed by desensitisation (e.g., Fig 3, panel (d)). For cases that presented a significant BP in both cILD and detection threshold, although no systematic general relation was observed, it was not uncommon to find similarities in their time patterns, as illustrated in Fig 3. Namely, some cases presented time-aligned mirror-inverse relations (e.g., panels (a) and (c)) while others showed symmetrical patterns (e.g., panels (b) and (d)). For all individual cILDs and thresholds, see Appendix (S1–S4 Figs).

**Fig 4:**
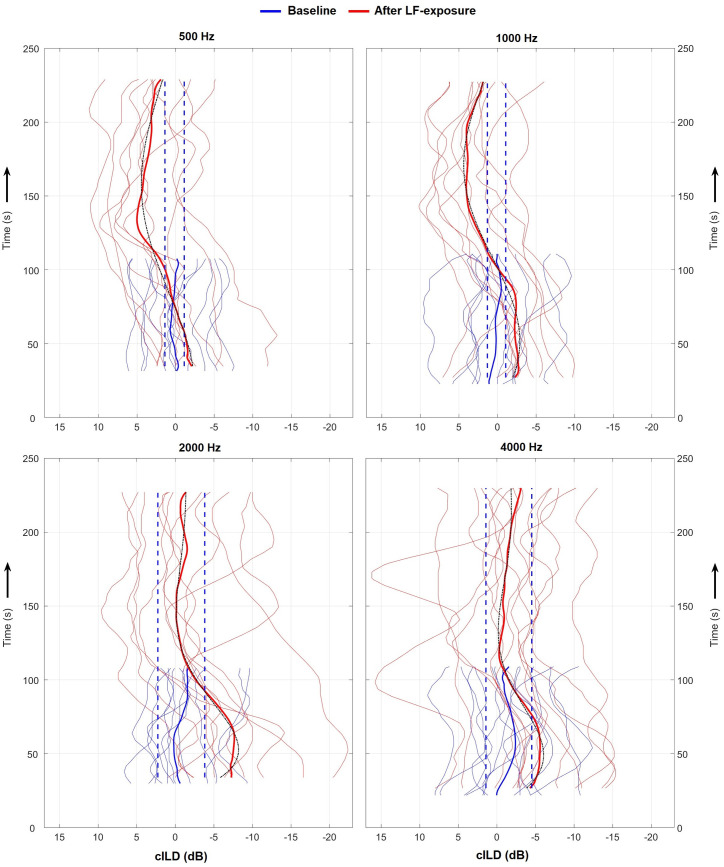
Individual and mean cILDs as a function of time obtained for each probe-tone frequency (upper labels). The blue and red curves correspond to conditions without and with a previous exposure to an intense low-frequency sound stimulus, respectively. Dashed blue lines show outlier limits of the average baseline cILDs (see data analysis for details). For each probe-tone frequency, only the subset of cases that passed the significance criterion have been included (N = 10, 10, 11 and 13 for *f* = 500, 1000, 2000 and 4000 Hz, respectively). Black dashed-dotted lines show harmonic oscillator fits to the average cILD of each probe-tone frequency.

Individual cILDs show that there was between-subject variation in the leftward or rightward offsets of the baseline cILDs as well as the cILDs obtained after LF-stimulation (Fig 4, thin lines). Generally, larger leftward or rightward baseline cILD offsets led to larger offsets in the corresponding direction for the LF-stimulation condition. The average level of each BP pattern was significantly positively correlated with the average baseline offset (considering all 44 valid BP cases: R^2^ = 0.75, *p *= 4.2 x 10^-14^). However, the mean baseline cILDs (thick blue) were relatively close to zero dB for each probe-tone frequency (across-time average levels: 0.2, 0.1, −0.7 and −1.5 dB for 0.5, 1, 2 and 4 kHz, respectively), due to the individual offsets nearly cancelling out in the averaging process. These offsets are an expected result of unequal sensitivities between both ears and are further addressed in the discussion. Despite these overall level differences, individual curves generally follow a similar trend of starting at relatively low negative cILDs (especially so for 2 and 4 kHz; with an individual case even < −20 dB) which progressively increase to then roughly flatten out.

Systematic variation in the BP pattern across probe-tone frequency, to some degree already observed in the individual examples shown in Fig 2, becomes most readily evident in the average cILDs of Fig 4 (red bold). The latter presented a time dependency that resembled that of a damped harmonic oscillator (black dashed lines), with clear deviations from the average baseline cILDs (see horizontal dashed lines delimiting the baseline outlier regions). For the two lower probe-tone frequencies, the average cILD transitioned from slightly negative values at the beginning (minimums of −2.1 and −2.7 dB for 0.5 and 1 kHz, respectively), into a positive region afterwards (with respective maximums of 5.0 and 4.0 dB; cILDs turned positive after about 100 s). It then slowly decayed, however, only approximating the baseline level range. This suggests the BP period for these probe-tone frequencies may have been longer than the 4-minute measurement duration, especially for 500 Hz. In contrast, for the two higher probe-tone frequencies the cILD was more markedly negative at the beginning (with minimums of −7.7 and −5.6 dB for 2 and 4 kHz, respectively), and then simply returned to baseline levels (with respective maximums of −0.1 and −0.3 dB), i.e., it did not cross the baseline range in the positive direction. The implied transition into a higher-damping oscillation as probe-tone frequency increased is confirmed by the oscillator model fit. The derived damping parameter (β) was indeed clearly higher for the two higher probe-tone frequencies than that for the two lower ones (β = 0.002, 0.003, 0.020 and 0.015 s^-1^ for 0.5, 1, 2 and 4 kHz, respectively). Further, the oscillation period (*T*) was found to decrease with increasing frequency (*T* = 278, 238, 186 and 167 s for 0.5, 1, 2 and 4 kHz), in line with the observation that the cILDs converged to baseline levels for the higher probe-tone frequencies at the measurement end, while not fully for the two lower probe-tone frequencies.

## IV. Discussion

Temporary changes in the ILD required to centre the sound image of binaurally presented pure tones were observed in normal-hearing participants after intense LF-sound exposure to one ear. It is unlikely that the observed cILD bias in the majority of recordings (65%) was due to attentional lapses or inconsistencies, as subjects received dedicated training, and the change-point analysis algorithm would have rejected their corresponding baseline cILDs. The majority of subjects (cILD: 98.5%, threshold: 81.3%) presented stable baselines that passed our criterion, which is supporting evidence that detected changes after the LF-sound stimulation were due to the BP. Further, in 61% of all recordings well-known BP patterns were observed in hearing threshold measurements. All of this supports the notion that observed cILD biases were due to the BP.

### A. Binaural signal conditions for a centre sound image

It has been shown previously that when the ITD is zero (as in our tests), pure tones presented via headphones at equal intensities (ILD = 0) will on average be perceived in the centre-of-the head position and when their intensities differ, the sound image will lateralise towards the ear stimulated at the higher intensity (demonstrated for pure tones with frequencies in the range 0.2–5 kHz in [38]; for further data, see review by [11]). However, other studies have shown that a more accurate condition for a centred sound image is provided when the tones are presented at equal sensation levels or at equal loudness ( [39,40]; equal sensation level has been also used to evaluate lateralisation of complex sounds [41]). For normal-hearing subjects presenting different pure-tone sensitivities between the left and right ears, it has been found that the sound image will be perceived in the centre-of-the head position if the tones are presented at equal sensation levels [40]. This is likely the reason why our baseline cILDs, although stable across time, presented individually different leftward or rightward offsets (interaural sensation-level differences cannot be reported as only the left-ear probe tone detection threshold was measured due to time constraints). Offsets from a centred sound image when the stimulus has zero dB ILD have also been observed in a more recent lateralisation study utilizing an alternative-forced choice paradigm [42], where sensation levels were not accounted for. Our offsets are also within 1.1 SDs of the distribution of hearing threshold differences between left and right ears (mean ≃ 0 dB, SD ≃ 9 dB) from a large sample study [43], and thus are explainable as due to unequal stimulus sensation levels between ears. Further, the ~ 24-dB ILD excursion span that we observed (equally for all probe-tone frequencies) matches well the ~ 24 dB span (~12 dB to each side of the midline to hear the stimulus completely on one side) obtained in [42] for 0.125-2-kHz tones, indicating subjects correctly tracked their PSL irrespective of observed offsets. As also nearly all participants (98.5%) were able to sustain a stable cILD baseline despite there being a larger or smaller systematic offset/bias, the latter are not thought to affect our BP hypothesis.

### B. On the physiological origins of the observed cILD bias

The observed cILD bias is consistent with the idea that peripheral sound transduction in the stimulated ear was affected by the LF-sound exposure, leading to changes in activity levels. The initially negative average cILDs after exposure (higher probe-tone levels on the right-hand side were required in order to keep the auditory image in the midline) indicate that the sound was biased to the left side, which would be expected if left-side activity levels increased. For the two probe tones with the lowest frequencies, the cILD then temporarily shifted into a positive region, i.e., the sound image was now biased to the right side. As the right-side tone level was fixed and the right ear was not exposed to the LF-sound, a right-side lateralisation can be expected if activity levels of the left-side probe tone now decreased. The observed cILD changes in both directions can be explained on a cochlear-mechanical level if the LF-sound exposure induced transient changes in the OP of the OHC MET, which affected activity levels in the stimulated left ear. These OP shifts, which exponentially decay towards the OP resting position in a pattern that may or may not oscillate [9,34,37], have been previously found to explain various BP patterns observed in hearing thresholds and OAE levels [4,34,44]. Using the 1^st^ derivative of the sigmoidal OHC MET transfer function as a measure of OHC efficiency [34], the various BP threshold patterns observed here and previously (including complex patterns of desensitisation-sensitisation and sensitisation-desensitisation) have been explained as due to the initial OP being below or above the MET inflection point [4]. For purposes of comparison with observed cILD patterns, different cILD and threshold patterns have been simulated (see Appendix, S5–S7 Figs). For suprathreshold sound processing, it was assumed that the OP shifted consistently towards one side of the sigmoidal MET, producing initial increases in OHC efficiency (leading to higher activity levels) on the stimulated left ear (S6 Fig). A different MET suprathreshold OP is consistent with studies indicating that the OP shifts with stimulus level [34,45–47]. Assuming the cILD is achieved when the relative left- and right-ear probe-tone levels are adjusted so that internal activity levels are equal in both ears [35,36], cILD patterns as observed here are readily explained by the model (S7 Fig). Whether under the BP the cILD crossed into a positive region depends here on the degree of damping of the OP shift, analogously as for threshold (examples of general trends observed for the lower and higher probe-tone frequencies are given in panels(a) and (b) of S7 Fig, respectively).

The fact that the measured cILD presented an oscillation with a larger period and less damping for the two lower probe-tone frequencies than the two higher ones (0.5 and 1 kHz vs 2 and 4 kHz), indicates that the lower-frequency probe tones were relatively more affected by exposure to the 30-Hz tone stimulus. In line with this, the number of cases where a BP could be identified for threshold was notably lower for the higher probe-tone frequencies. These observations agree with those of previous studies on the BP [4,44] and might be a consequence of cochlear tonotopy: OHCs located at apical places will be much more displaced by a low-frequency tone than those located at basal places, as the basilar membrane displacement gradient by low-frequency tones has been estimated to be 9 dB/octave towards its base [48].

### C. Implications for sound localisation

Binaural processing of ILDs and ITDs is the major basis for sound localisation in the horizontal plane [35], and enhances speech comprehension by segregating different sound sources into auditory streams [12]. Although cILDs were measured with earphones in this study, they have implications for spatial sound localisation, which relies strongly on these cues [10]. Observed cILD bias excursions for the average curves (7.1, 6.7, 7.5 and 5.3 dB for 0.5, 1, 2 and 4 kHz, respectively) are expected to produce azimuthal shifts ~41° to 69° in the source image position (according to the approximate expression: $ILD=0.18\;\sqrt{f}\sin(\theta$) [49]). Considering that even larger cILD biases were not uncommon in individual cases, it can be expected that sound source localisation will be severely biased after intense low-frequency sound exposure.

Localisation errors associated to the cILD biases that we observed are in qualitative agreement with the generally poorer sound localisation performance observed in the hearing impaired [50]. Inducing the BP on a single ear (as we did) is perhaps most relatable to the degradation of ILD cues that occurs in asymmetric or unilateral hearing losses (with the unstimulated ear serving initially as the ear with reduced activity relative to the stimulated ear), which have been found to produce the poorest localisation performance amongst hearing losses [50]. Further, sound localisation experiments have shown that horizontal-plane localisation is largely biased towards the open ear when an asymmetrical conductive loss is simulated by plugging one ear of normal-hearing listeners (on average by ~31° in [51]; individual biases of up to 56° are reported in [16]). From this perspective, the LF-stimulated ear in our study acted first as the “better ear”, and later (at least for the two lower probe-tone frequencies) as an ear with cochlear/conductive hearing loss relative to the unexposed ear, before returning to normal state. The induced cILD biases that we observed translate to comparable or even larger lateral shifts in the sound source image than that reported by previous studies occluding one ear. It has been previously found that awareness of such procedures by tested subjects may have strong influences on the activation of compensatory mechanisms, as evident from a number of animal studies showing fast reweighting of binaural cues (reviewed in [52]). However, since adaptation to deteriorated binaural cues has been found to take at least a few hours in normal-hearing listeners with simulated losses [52], the transient nature of the BP makes them irrelevant.

It should be noted that in the case of environmental, sound field LF-sound exposure, the BP might affect both ears, so that interaction between the left- and right-ear BPs may lead to even larger distortions in ILD processing. The latter may occur if the left- and right-ear BPs are not aligned in their time course (however, if they happened to be precisely aligned, the effect might get cancelled out, as both ears would undergo the same time-variant bias). Moreover, it should also be taken into account that alteration of binaural processing as we observed is not only expected to affect sound localisation, but it might also affect the ability to unmask signals from spatially separated sound sources [52,53]. Recent studies suggest that the mammalian binaural auditory system, in fact, did not evolve for accurate localisation of sound sources in complex acoustic situations but rather for sound segregation. The neural structures originally processing ILD [54] and ITD [55] adapt rapidly via negative feedback circuits. This leads to surprisingly strong systematic localisation errors, but at the same time also to improved sound source segregation, as has been shown in human subjects [56]. Mammals (including humans), therefore, do not appear to be absolute, but rather relative sound localisers. The present study therefore represents proof of principle that the BP can bias the perceived lateral position of sound sources under controlled experimental conditions. The consequences of fluctuating thresholds and altered ILD processing as in the BP for our daily life (e.g., verbal communication) require further studies using more complex and natural test situations.

## V. Summary

Exposure to intense low-frequency sound often produces transient biases in ILD processing, which largely impacts sound lateralisation and in turn can be expected to deteriorate sound localisation ability.

## Supporting information

S1 Fig Individual interaural level differences to centre a sound source image (cILD) and detection thresholds. The cILDs obtained for the 500-Hz probe tone are shown both before (blue) and right after (red) stimulation by an intense low-frequency stimulus (30-Hz tone at 120 dB SPL, 90 s). The horizontal blue dashed lines are lower and upper outlier limits based on the baseline distribution. Black lines are detection thresholds obtained right after the same stimulation, in a separate measurement. To facilitate comparison, all curves have been normalized by their corresponding mean baseline level. Significance markers are given in the upper left and are as follows. Filled/unfilled larger upper circles: BP valid/invalid; filled/unfilled smaller lower circles: a BP was/was not observed in the LF-stimulation condition (in both cases, red circles: cILD, black circles: threshold); Checkmark (✓) and cross (x) indicate whether the baseline was stable or not (blue: cILD, black: threshold). Note that if the baseline did not pass our statistical criterion, a BP observed in cILD/threshold was not deemed as valid. The upper-right numbers are subject identifiers and are the same for data on all other probe frequencies shown below. For further detail, see methods.
(TIF)

S2 Fig Same as S1 Fig, for the 1000-Hz probe frequency. (TIF)

S3 Fig Same as S1 Fig, for the 2000-Hz probe frequency. (TIF)

S4 Fig Same as S1 Fig, for the 4000-Hz probe frequency. (TIF)

S5 Fig Prediction of OHC efficiency changes from a qualitative model of the OHC MET (as in [4]). (a) Sigmoidal MET input/output function (a Boltzmann function as in [4,57], arbitrarily centered at its inflection point). The blue and red dots represent initial operating point positions below and above the inflection point, respectively. Vertical lines show the range of the operating point shift (that starts in the hyperpolarizing direction) around each initial operating point, for two oscillation examples (underdamped: dashed lines; nearly critically damped: dotted lines). (b) Operating point shifts as a function of time, for the two oscillation examples (solid: underdamped; dotted: nearly critically damped). (c) Derivative of the input-output function, representing OHC efficiency [34]. Operating point positions and shifts are similarly given as in (a); (d) Changes in OHC efficiency as function of time resulting from the operating point shifts. The qualitative model can account for the different bounce patterns observed in threshold, assuming operating points can be located both below (blue) and above (red) the MET inflection point.
(TIF)

S6 Fig As S5 Fig, assuming initial operating point positions are only on one side of the OHC MET inflection point (arbitrarily to the right). Also similarly, two operating point shifts have been simulated (with damping coefficients β_1_ and β_2_, where β_1 _< β_2_; and periods *T*_1_ and *T*_2_, where *T*_1_ > *T*_2_), simulating average trends observed for the lower and upper probe frequencies (blue and red lines, respectively).
(TIF)

S7 Fig Simulated centering ILD (cILD) patterns. These are shown for the two operating point shifts described in S6 Fig (examples for the lower and higher probe-tone frequencies are given in panels (a) and (b), respectively). It has been assumed that: (1) Activity levels increased/decreased in the stimulated left ear from transient changes in OHC efficiency (S6 Fig. d). (2) Activity levels across time in the right ear remained the same; (3) Across time, the left- and right-ear input sound levels (L_left_ and L_right_, respectively) were adjusted to produce equal (internal) activity levels to reach a centred sound source image, with their difference determining the simulated cILD (upper red axis). In panel (a) it has been assumed that the suprathreshold stimulus has equal sensation levels in both ears, while in panel (b) it was assumed that the sensation level in the right ear was 2 dB lower and that this led to a 2-dB decrease in right-ear activity levels.
(TIF)

S1 Data ILD and Threshold Data. (ZIP)
