# Supplementary figures and images for: Intense low-frequency sound transiently biases human sound lateralisation
Source: PLoS One. 2025 Jun 30;20(6):e0327525. doi: 10.1371/journal.pone.0327525 (PMC12208446; doi:10.1371/journal.pone.0327525)

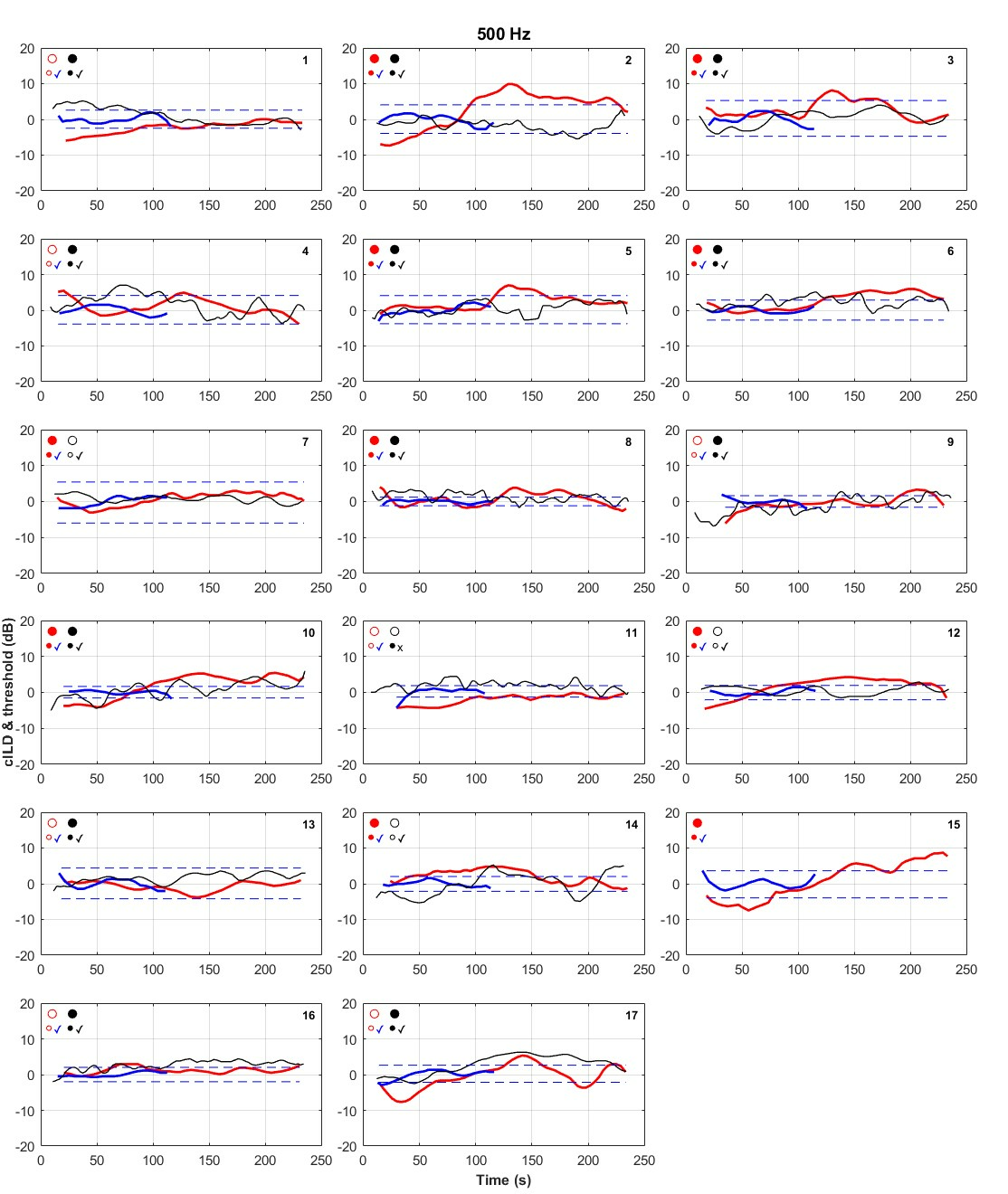

Supplement: S1 Fig — The cILDs obtained for the 500-Hz probe tone are shown both before (blue) and right after (red) stimulation by an intense low-frequency stimulus (30-Hz tone at 120 dB SPL, 90 s). The horizontal blue dashed lines are lower and upper outlier limits based on the baseline distribution. Black lines are detection thresholds obtained right after the same stimulation, in a separate measurement. To facilitate comparison, all curves have been normalized by their corresponding mean baseline level. Significance markers are given in the upper left and are as follows. Filled/unfilled larger upper circles: BP valid/invalid; filled/unfilled smaller lower circles: a BP was/was not observed in the LF-stimulation condition (in both cases, red circles: cILD, black circles: threshold); Checkmark (✓) and cross (x) indicate whether the baseline was stable or not (blue: cILD, black: threshold). Note that if the baseline did not pass our statistical criterion, a BP observed in cILD/threshold was not deemed as valid. The upper-right numbers are subject identifiers and are the same for data on all other probe frequencies shown below. For further detail, see methods. (TIF) [file pone.0327525.s001.tif]

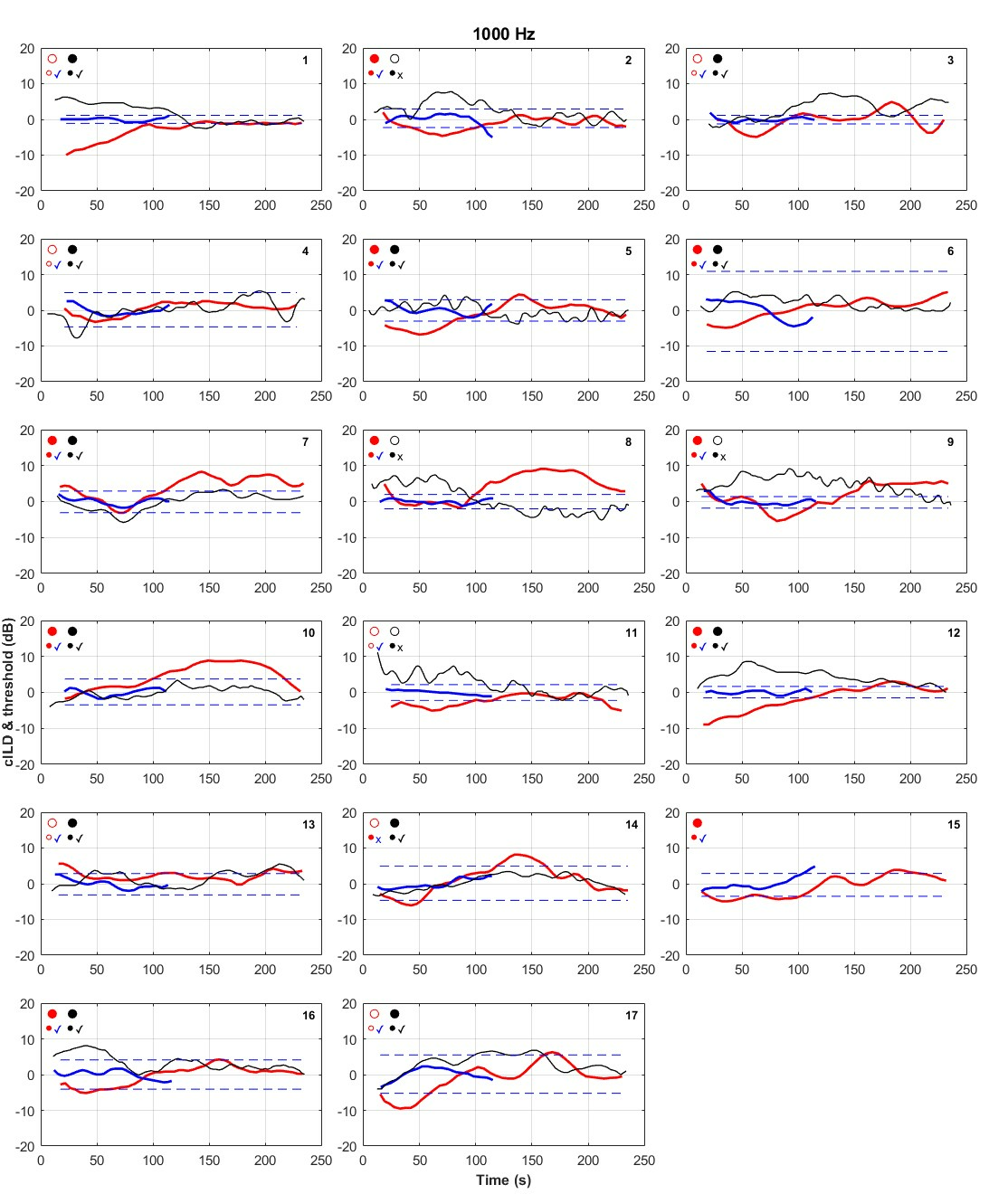

Supplement: S2 Fig — (TIF) [file pone.0327525.s002.tif]

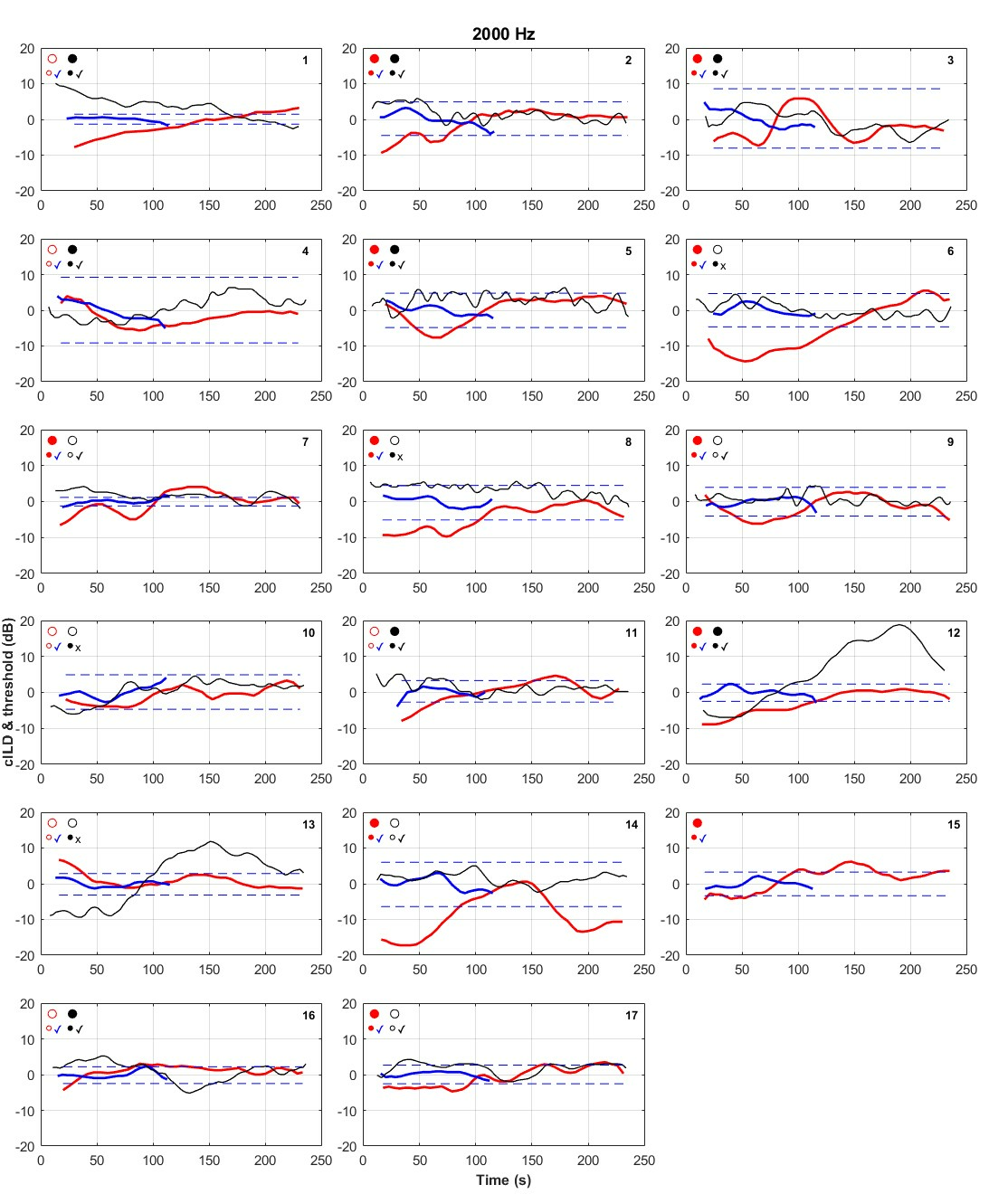

Supplement: S3 Fig — (TIF) [file pone.0327525.s003.tif]

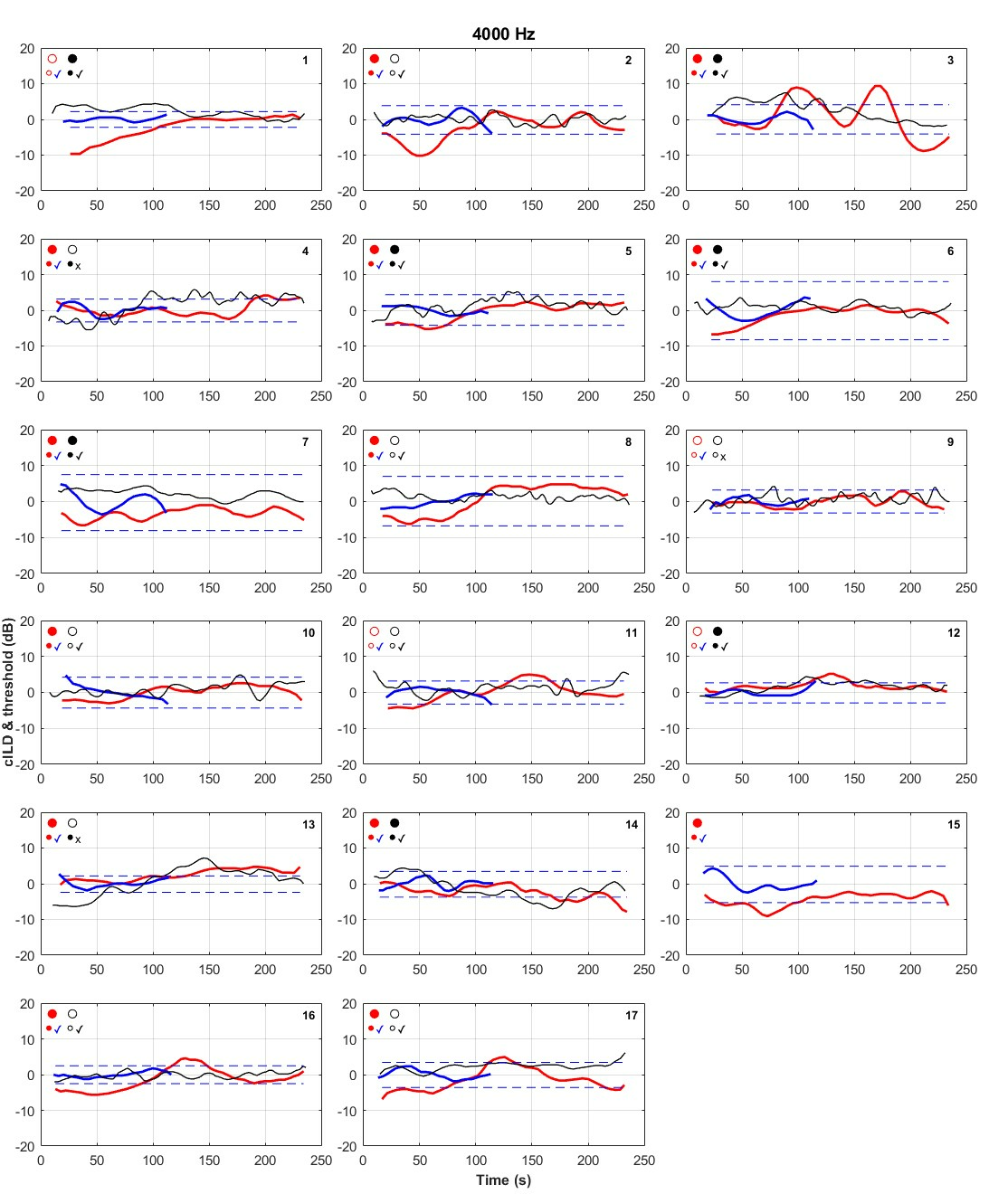

Supplement: S4 Fig — (TIF) [file pone.0327525.s004.tif]

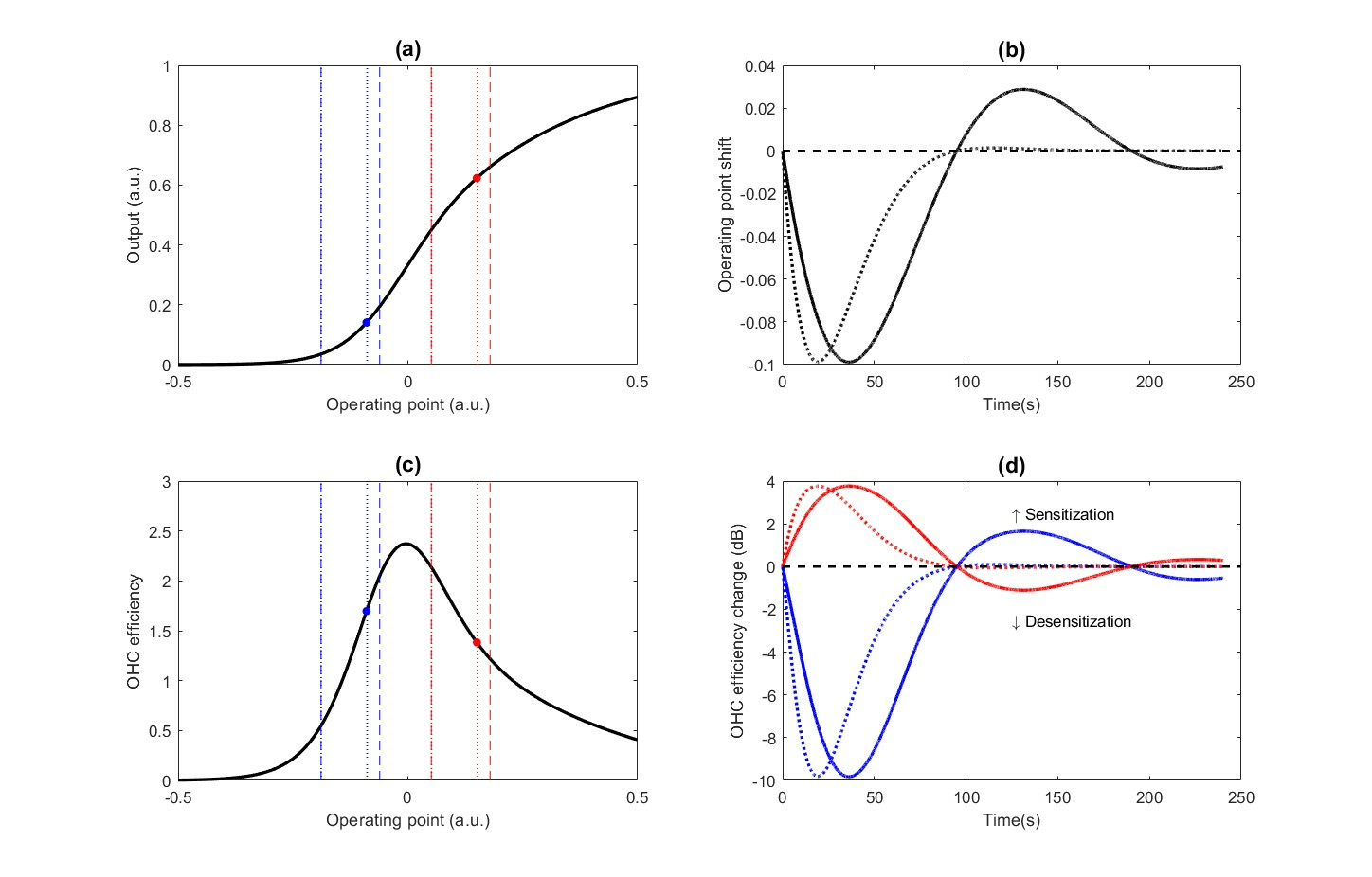

Supplement: S5 Fig — (a) Sigmoidal MET input/output function (a Boltzmann function as in [4,57], arbitrarily centered at its inflection point). The blue and red dots represent initial operating point positions below and above the inflection point, respectively. Vertical lines show the range of the operating point shift (that starts in the hyperpolarizing direction) around each initial operating point, for two oscillation examples (underdamped: dashed lines; nearly critically damped: dotted lines). (b) Operating point shifts as a function of time, for the two oscillation examples (solid: underdamped; dotted: nearly critically damped). (c) Derivative of the input-output function, representing OHC efficiency [34]. Operating point positions and shifts are similarly given as in (a); (d) Changes in OHC efficiency as function of time resulting from the operating point shifts. The qualitative model can account for the different bounce patterns observed in threshold, assuming operating points can be located both below (blue) and above (red) the MET inflection point. (TIF) [file pone.0327525.s005.tif]

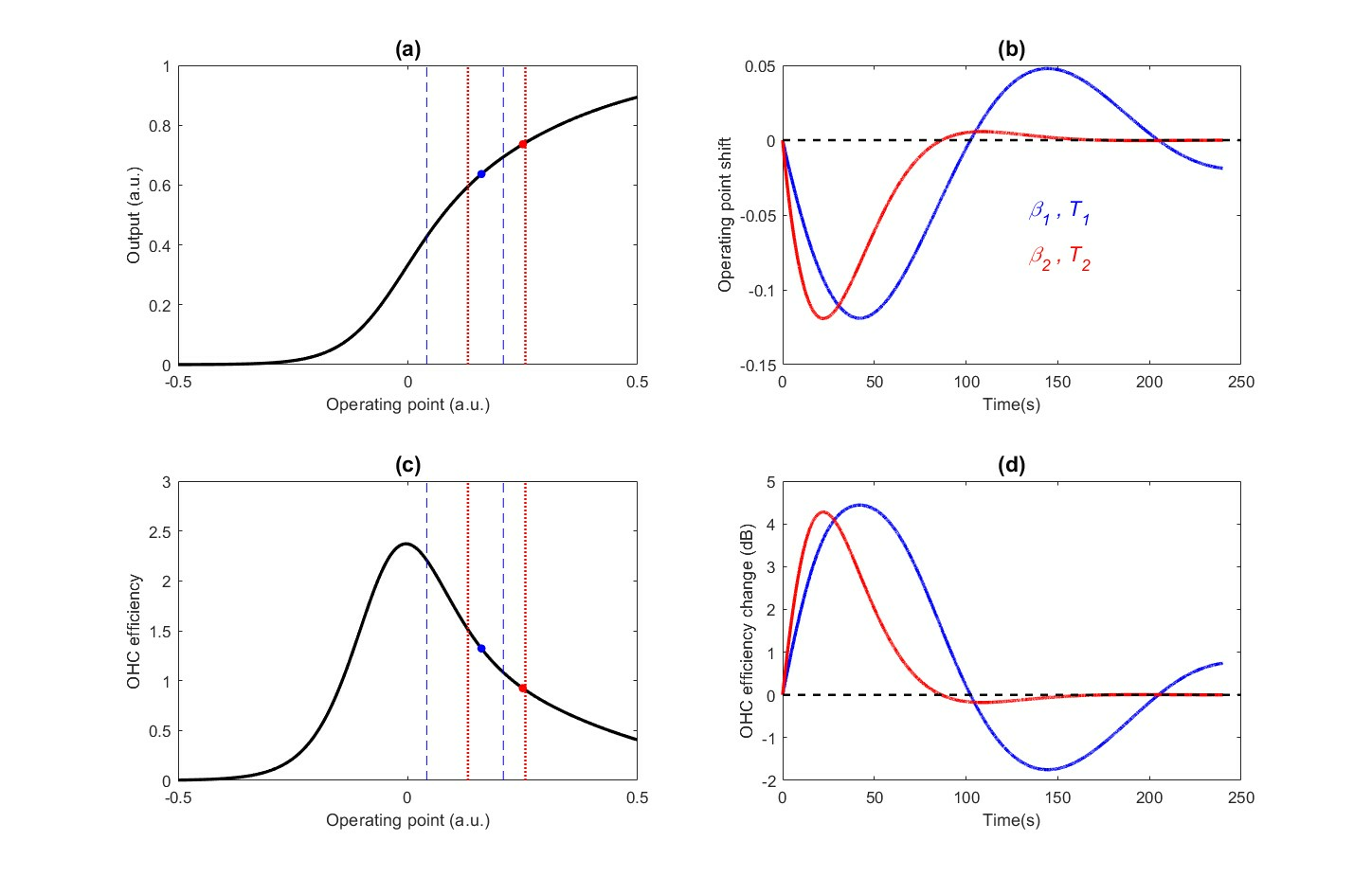

Supplement: S6 Fig — Also similarly, two operating point shifts have been simulated (with damping coefficients β1 and β2, where β1 < β2; and periods T1 and T2, where T1 > T2), simulating average trends observed for the lower and upper probe frequencies (blue and red lines, respectively). (TIF) [file pone.0327525.s006.tif]

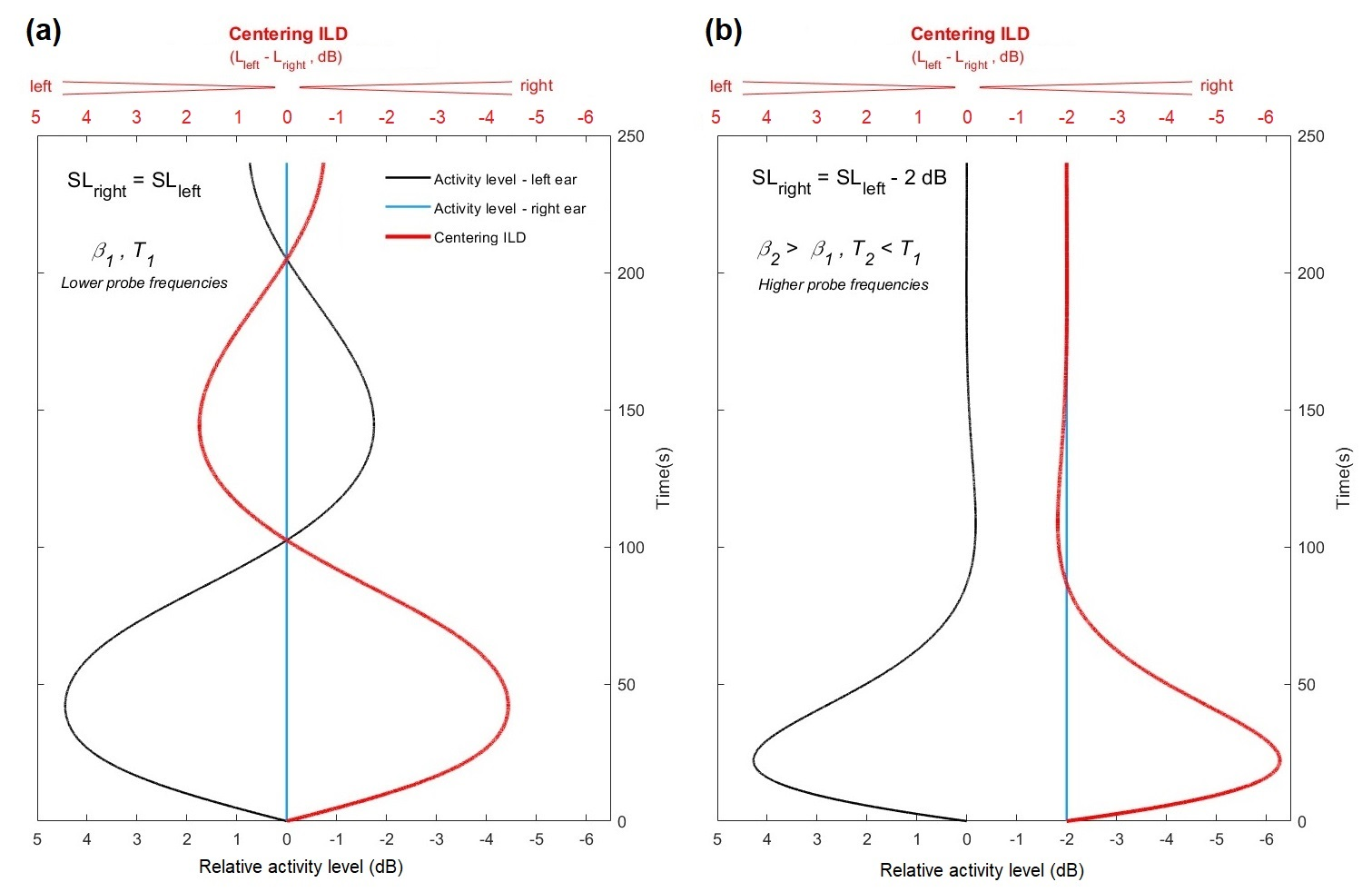

Supplement: S7 Fig — These are shown for the two operating point shifts described in S6 Fig (examples for the lower and higher probe-tone frequencies are given in panels (a) and (b), respectively). It has been assumed that: (1) Activity levels increased/decreased in the stimulated left ear from transient changes in OHC efficiency (S6 Fig. d). (2) Activity levels across time in the right ear remained the same; (3) Across time, the left- and right-ear input sound levels (Lleft and Lright, respectively) were adjusted to produce equal (internal) activity levels to reach a centred sound source image, with their difference determining the simulated cILD (upper red axis). In panel (a) it has been assumed that the suprathreshold stimulus has equal sensation levels in both ears, while in panel (b) it was assumed that the sensation level in the right ear was 2 dB lower and that this led to a 2-dB decrease in right-ear activity levels. (TIF) [file pone.0327525.s007.tif]
